# Iridoids, Phenolic Compounds and Antioxidant Activity of Edible Honeysuckle Berries (*Lonicera caerulea* var. *kamtschatica* Sevast.)

**DOI:** 10.3390/molecules22030405

**Published:** 2017-03-05

**Authors:** Alicja Z. Kucharska, Anna Sokół-Łętowska, Jan Oszmiański, Narcyz Piórecki, Izabela Fecka

**Affiliations:** 1 Department of Fruit, Vegetable and Plant Nutraceutical Technology, Wrocław University of Environmental and Life Science, Chełmońskiego 37, 51-630 Wrocław, Poland; anna.sokol-letowska@upwr.edu.pl (A.S.-L); jan.oszminski@upwr.edu.pl (J.O.); 2 Arboretum and Institute of Physiography in Bolestraszyce, 37-700 Przemyśl, Poland; narcyz360@gmail.com; 3 University of Rzeszów, Towarnickiego 3, 35-959 Rzeszów, Poland; 4 Department of Pharmacognosy, Wrocław Medical University, Borowska 211A, 50-556 Wrocław, Poland; izabela.fecka@umed.wroc.pl

**Keywords:** honeysuckle berries, UPLC-ESI-qTOF-MS/MS, iridoids, 8-*epi*-loganic acid, phenolic compounds, taxifolin, cultivars, genotypes, antioxidant activity

## Abstract

Iridoid and polyphenol profiles of 30 different honeysuckle berry cultivars and genotypes were studied. Compounds were identified by ultra-performance liquid chromatography coupled with electrospray ionization mass spectrometry (UPLC-ESI-qTOF-MS/MS) in positive and negative ion modes and quantified by HPLC-PDA. The 50 identified compounds included 15 iridoids, 6 anthocyanins, 9 flavonols, 2 flavanonols (dihydroflavonols), 5 flavones, 6 flavan-3-ols, and 7 phenolic acids. 8-*epi*-Loganic acid, pentosyl-loganic acid, taxifolin 7-*O*-dihexoside, and taxifolin 7-*O*-hexoside were identified in honeysuckle berries for the first time. Iridoids and anthocyanins were the major groups of bioactive compounds of honeysuckle constituents. The total content of quantified iridoids and anthocyanins was between 128.42 mg/100 g fresh weight (fw) (‘Dlinnoplodnaya’) and 372 mg/100 g fw (‘Kuvshinovidnaya’) and between 150.04 mg/100 g fw (‘Karina’) and 653.95 mg/100 g fw (‘Amur’), respectively. Among iridoids, loganic acid was the dominant compound, and it represented between 22% and 73% of the total amount of quantified iridoids in honeysuckle berry. A very strong correlation was observed between the antioxidant potential and the quantity of anthocyanins. High content of iridoids in honeysuckle berries can complement antioxidant properties of phenolic compounds.

## 1. Introduction

Edible honeysuckle berries (*Lonicera caerulea* L. var. *kamtschatica* Sevast.; Caprifoliaceae family) are gaining popularity in many European countries such as Russia, Poland, the Czech Republic, and others. The attractiveness of these fruits derives from a number of factors, including early time of ripening (in Poland before strawberries), resistance to spring frost, flavor, high contents of vitamin C and polyphenol compounds, and health-related properties. The fruits are good plant material for the food industry, for the production of juices, jams, purees, and—for the pharmaceutical industry—for the production of food supplements [1,2,3].

The health benefits of honeysuckle berries have been known and used for a long time in traditional medicine in Russia and China. Current research, both in vitro and in vivo, also supports the traditional medical use of honeysuckle berries. Recent studies indicate, among others, the antioxidant, anti-inflammatory and antibacterial properties of the extract from honeysuckle berry fruits [4,5,6]. The authors explain the health benefits of honeysuckle berries by the occurrence in the fruit of polyphenol compounds, mainly glycosides of anthocyanins. In addition to the anthocyanins, phenolic acids, flavonols, flavones and flavan-3-ols are also present [3,4,7,8]. Their contents vary in fruits, and depend on many factors, including cultivar and genotypes [9,10,11,12,13,14,15]. In addition to the polyphenols, in honeysuckle berries iridoids have also been identified [16,17,18,19]. Among the 13 iridoids we have identified epimeric pairs of loganic acid and loganin, sweroside, secologanin, secoxyloganin, and additionally pentosides of loganic acid (two isomers), pentosides of loganin (three isomers), and pentosyl-sweroside [19].

In Russia, Poland, the Czech Republic, Canada, Japan, and other countries, many honeysuckle cultivars have been selected. They differ in the content of bioactive compounds, appearance (size, shape), time of ripening, yield from the bush, growing conditions, and taste. Cultivar diversity is great, because of the search for a cultivar with large and tasty fruits. Honeysuckle berry fruits are sweet and sour, with a somewhat bitter aftertaste, and resemble blackcurrants and blueberries.

In the fruits, sugars affect the degree of sweetness, while organic acids are responsible for the sour taste. The tartness of fruit is determined by the quantity and quality of polyphenols, whereas the bitterness, among other compounds, is determined by secoiridoids [20]. In addition to the fact that polyphenol and iridoid compounds may affect the taste of the fruit, they exhibit high biological activity [21]. Many authors indicate their antioxidant and anti-inflammatory properties [5,22,23,24,25]. Iridoids, in contrast to the polyphenols, are rarely found in fruits. Exceptions include cornelian cherry fruits [26], cranberry [27], bilberry [28], and, recently investigated by us, honeysuckle berries [17,18,19]. In this study, we determined the content of loganic acid, loganin, their three derivatives, and the quantity of iridoids in berries of honeysuckle. There are no publications on the quantitative analysis of different iridoids such as loganin 7-*O*-pentoside or loganic acid 7-*O*-pentoside in honeysuckle berries. Therefore, the aim of this study was qualitative and quantitative determination of iridoids and also of polyphenols in berries of 27 cultivars and 3 genotypes of blue honeysuckle (*L. caerulea* var. *kamtschatica*) with the usage of chromatographic methods, as well as the determination of their antioxidant activity.

## 2. Results and Discussion

### 2.1. Qualitative Identification of Iridoids and Phenolic Compounds

The results of qualitative identification of the compounds of honeysuckle berries are presented in Table 1, Figures S1 and S2. The compounds were identified by their UPLC retention times, elution order, spectra of the individual peaks (UV/Vis, MS), spectral data and by comparison with literature data. In our research, we determined 50 compounds from two groups: monoterpenes (iridoids) and polyphenols (anthocyanins, flavonols, flavanonols, flavones, flavan-3-ols, phenolic acids).

Among the compounds of the first analyzed group, we identified iridoids. In our previous initial studies, we determined thirteen iridoids (loganic acid (LA), loganin (Lo), sweroside (S), their derivatives, and epimeric pairs of LA and Lo) from honeysuckle berries [17,18,19]. In this study, fifteen compounds with typical UV/Vis spectra were discovered. Among them, compounds **3** and **12** were identified in honeysuckle berries for the first time. In the former, higher abundance was observed for the ion at *m*/*z* 375.1276 [M − H]^−^ in negative electrospray ionisation (ESI) mode than for the ion at *m*/*z* 377.1440 [M + H]^+^ in positive ESI mode (Figure S3). This compound (*t*_R_ 2.80 min) displayed the same pseudomolecular and fragment ions as LA (compound **9**; *t*_R_ 3.73 min) and 7-*epi*-LA (compound **13**; *t*_R_ 4.27 min), but they differed in the retention times and abundance of the major fragment ions. In compound **3**, high abundance was observed for ions at *m*/*z* 213.0769 [M − 162 − H]^−^, similarly as in LA, and 197.0802 [M − 162 − 18 + H]^+^, similarly as in 7-*epi*-loganic acid (7-*epi*-LA) (Figure S3). In the previous study, we showed that for 7-*epi*-LA, ion [M − 162 − H]^−^ was less stable in negative ESI mode, when the –OH group in C-7 is below the molecule plane; therefore the ion appears after loss of water [M – 162 − 18 − H]^−^ [19]. It is similar in the case of 8-*epi*-loganic acid (8-*epi*-LA) in positive ESI mode. Ion [M − 162 + H]^+^ is less stable when the –CH_3_ group in C-8 is below the molecule plane; therefore the ion appears after loss of water [M − 162 − 18 + H]^+^. There are no reports about the contents of this compound in other fruits. 8-*epi*-LA and its derivatives were isolated only from green plants [29,30]. The second newly identified iridoid was compound **12**. In negative mode, this compound had a pseudomolecular ion at *m*/*z* 507.1746 [M − H]^−^ and fragment ions at *m*/*z* 375.1356 [M − 132 − H]^−^, 213.0769 [M − 132 − 162 − H]^−^, and 169.0855 [M − 132 − 162 − 44 − H]^−^. So this was identified tentatively as a pentose derivative of loganic acid. Similarly to pentosyl-loganin (pLo) [19], this compound did not show ions [M − 162 − H]^−^ and [M − 162 − 18 − H]^−^. According to these data, in compound **12**, pentose is attached to glucose, producing a disaccharide similar to compound **24**. It seems that this is pentosyl-loganic acid (pLA) (**12**).

Among the compounds of the second determined group (polyphenols), we identified basic anthocyanins, flavonols, flavanonols (dihydroflavonols), flavones, flavan-3-ols, and phenolic acids. Six anthocyanins were identified in positive mode. Compounds **10**, **17**, and **21** exhibited pseudomolecular ions [M + H]^+^ at *m*/*z* 611.1664, 449.1107, and 595.1664 and a similar fragment ion at *m*/*z* 287.0536, which corresponded to the molecule of the cyanidin aglycone, after loss of a diglucose, glucose, and rutinose, respectively. Compound **23** gave a fragment ion at *m*/*z* 271.0601 after loss of 162 Da, which corresponded to the pelargonidin 3-*O*-glucoside (Pg 3-glc), whereas compounds **28** and **31** showed a fragment ion at *m*/*z* 301.1730 after loss of 162 and 308 Da, which corresponded to the peonidin 3-*O*-glucoside (Pn 3-glc) and peonidin 3-*O*-rutinoside (Pn 3-rut), respectively. These results agreed with recently published data [3,4].

**Figure 1:**
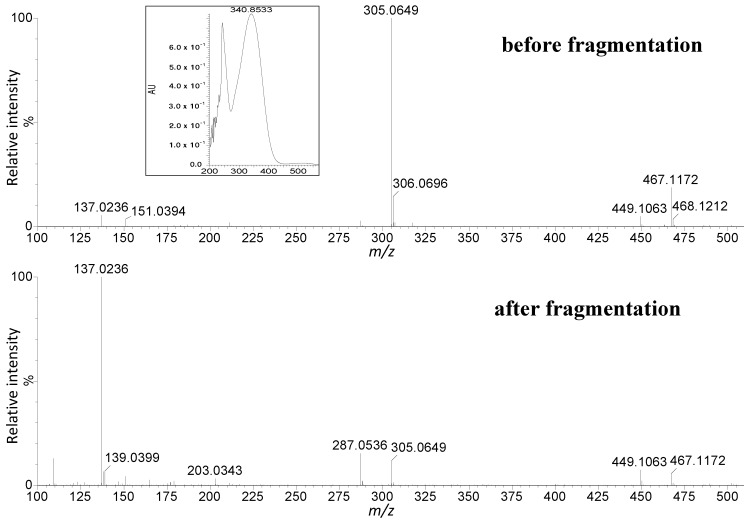
Mass spectra of the ions of taxifolin 7-*O*-hexoside in positive mode before and after fragmentation and UV/Vis spectra.

Among the nine flavonols, there were seven derivatives of quercetin (compounds **30**, **34**, **36**, **38**, **39**, **40**, **43**, **48**) with an aglycone ion at *m*/*z* 301.0354 and isorhamnetin (compound **45**) with an aglycone ion at *m*/*z* 315.0512. In this study, one compound (**30**) at *m*/*z* 625.1386 [M − H]^−^, two compounds (**34**, **36**) at *m*/*z* 595.1312 [M − H]^−^, two compounds (**38**, **39**) at *m*/*z* 609.1483 [M − H]^−^, one compound (**40**) at *m*/*z* 463.0887 [M − H]^−^, and one compound (**48**) at *m*/*z* 505.0979 [M − H]^−^ were found. Compound **45** has pseudomolecular ions at *m*/*z* 609.1483 [M − H]^−^ and 447.0916 [M − 162 − H]^−^, which corresponded to isorhamnetin hexosyl-pentoside. These results are consistent with previous studies [3]. Besides flavonols, we identified in honeysuckle berries also two flavanonols (dihydroflavonols) for the first time. Their UV/Vis and mass spectra in positive mode are shown in Figure 1. Compounds **4** and **14** exhibited pseudomolecular ions [M + H]^+^ at *m*/*z* 629.1733 and 467.1173, respectively. They have similar fragment ions at *m*/*z* 305.0649 and 287.0536, which correspond to the aglycone of taxifolin and taxifolin after the water loss [Aglycone − 18 + H]^+^. In the positive mode both taxifolin glycosides beside pseudomolecular ions [M + H]^+^ at 629.1733 and 468.1172 provided additional ions at 611.1614 and 449.1063 corresponding to structures about 18 Da smaller [M − 18 + H]^+^ after the loss of water. This observation confirms that compounds **4** and **14** have free –OH at the C-3 position, and they are probably taxifolin 7-*O*-dihexoside (**4**) and 7-*O*-hexoside (**14**), respectively. The most probable structures of fragment ions of taxifolin derivatives in positive mode are shown in Figure S4 and Table S1. Flavanonols have been detected for example in *Rosa canina* and *R. micrantha* fruits [31] but not in blue honeysuckle berries.

Five compounds belonging to flavones were also detected. They were four derivatives of luteolin (compounds **15**, **41**, **42**, **44**) with an aglycone ion at *m*/*z* 285.0394 [M − H]^−^ and diosmetin (compound **50**) with an aglycone ion at *m*/*z* 299.0536 [M − H]^−^. In previous studies Oszmiański et al. [3] identified four derivatives of luteolin. Compound **15** (*t*_R_ 4.44 min) had a pseudomolecular ion at *m*/*z* 771.2018 [M − H]^−^ and fragment ions at *m*/*z* 609.1432 [M − 162 − H]^−^, 447.0959 [M − 162 − 162 − H]^−^, and 285.0394 [M − 162 − 162 − 162 − H]^−^. It was identified tentatively as luteolin trihexoside. Compounds **41** and **44** gave the same pseudomolecular ion at *m*/*z* 593.1507 [M − H]^−^ but they were different in the retention time. Compounds **41** and **44** had two fragment ions at *m*/*z* 447.0918 [M − 146 − H]^−^ and 285.0394 [M − 146 − 162 − H]^−^. These compounds are luteolin 7-*O*-rutinoside and luteolin *O*-deoxyhexosyl-hexoside, which is consistent with earlier studies [3]. Compound **42** was identified as luteolin 7-*O*-glucoside, with a pseudomolecular ion at *m*/*z* 447.0918 and a fragment ion at 285.0394 obtained after the loss of 162 Da, which has been confirmed by other authors [3,7] and compared with data for the proper standard. Compound **50** was identified as 7-*O*-rutinoside of diosmetin (diosmin) when compared with the standard.

Among the flavan-3-ols, we identified six compounds: (+)-catechin (**6**) and five oligomeric procyanidins type B—two PC dimers (**2**, **11**), two PC trimers (**5**, **20**), and a PC tetramer (**22**), which had characteristic pseudomolecular (compound **6**), or fragment (compounds **2**, **5**, **11**, **20**, **22**) ions at *m*/*z* 289.0723 and others [32]. Oszmiański et al. [3], in addition to these compounds, also identified (–)-epicatechin. Seven phenolic acids including three caffeoylquinic acids (compounds **1**, **8**, **18**) with a fragment ion at *m*/*z* 191.0553, three dicaffeoylquinic acids (compounds **46**, **47**, **49**) with fragment ions at m/z 353.0879 and 191.0553 and one caffeoylglucose (compound **7**) with a fragment ion at *m*/*z* 179.0349, were identified, which was consistent with the data reported by other authors [3].

### 2.2. Quantitative Identification of Iridoids and Phenolic Compounds

Fifty compounds from the iridoid and polyphenolic groups were identified with the UPLC-qTOF-MS/MS method (Table 1), but only major compounds were quantified using HPLC-PDA detection (Table 2, Table 3, Table 4, Table 5, Table 6 and Table 7). Quantitative analysis of constituents of honeysuckle berry was performed for 27 cultivars and 3 genotypes.

The qualitative and quantitative compositions of iridoids were very different for all cultivars and genotypes. Five of the 14 identified iridoids, i.e., 8-*epi*-LA, pLo, pentosyl-sweroside (pS), 7-*epi*-loganin (7-*epi*-Lo), and secoxyloganin (secoxyLo), were present in trace amounts in the studied berries. The content of a further nine iridoids in fresh honeysuckle berries is presented in Table 2. Four of them (LA, 7-*epi*-loganic acid 7-*O*-pentoside (7-*epi*-LAp), loganin (Lo), and sweroside (S)) were present in all cultivars and genotypes, but there were differences in their levels: LA > Lo + S > 7-*epi*-LAp. Five other iridoids (7-*epi*-LA, loganic acid 7-*O*-pentoside (LAp), loganin 7-*O*-pentoside (Lop), 7-*epi*-loganin 7-*O*-pentoside (7-*epi*-Lop), and secologanin (secoLo)) were present in some cultivars only. The total quantified iridoid content in honeysuckle berries covered a wide range from 119.95 mg/100 g fresh weight (fw) (‘Dlinnoplodnaya’) to 276.43 mg/100 g fw (‘Berry Smart Blue’). The average total content of quantified iridoids, evaluated by HPLC analysis, was 180.77 mg/100 g fw. We previously observed higher, in terms of the average value, total content of quantified iridoids in cornelian cherry fruits and honeysuckle berry [18,26]. Contents of LA and its derivatives were more than twice the content of Lo, S, and their derivatives. In honeysuckle berries, the dominant compound among iridoids was LA (mean 81.09 mg/100 g fw). The lowest amount of this compound (35.22–39.21 mg/100 g fw) was found in ‘Vostorg’, ‘Sineglazka’, and ‘Chelyabinka’, whereas the highest (182.28 mg/100 g fw) was found in ‘Kuvshinovidnaya’. LA represented between 22% and 73% of the total content of quantified iridoids in honeysuckle berry. This range is very wide and depends strongly on the cultivar or genotype. In our previous studies, we investigated LA content in many cornelian cherry cultivars, but the percentage content of iridoid in these fruits was in a narrower range, i.e., 88%–96% [26]. The high LA content is beneficial, because this compound exhibits biological activity. Many authors have shown that LA has strong anti-inflammatory properties [33,34,35]. In studies in rabbits Sozański et al. [34,35] revealed that LA diminished diet-induced dyslipidemia and atherosclerosis, increased PPAR-*α* and PPAR-γ expression, and exhibited anti-inflammatory activity. In the ten cultivars of analyzed berries, there was determined the *epi*-isomer of LA, which constituted 11% of the total amount of quantified iridoids, on average. The highest amount of this compound (45.05 mg/100 g fw) was present in ‘Viola’. *epi*-LA, like LA, also exhibits biological activity. According to Dinda et al. [36], *epi*-LA exhibited strong antibacterial activity against *Escherichia coli* and *Staphylococcus aureus*.

LAp and 7-*epi*-LAp were the two major derivatives of isomers of LA, but the first compound was about three times more abundant than the second. Concentrations of LAp ranged from 9.37 mg/100 g fw in “Blue Velvet” to 71.16 mg/100 g fw in ‘Kamchadalka’. This compound was identified in 24 cultivars, excluding ‘Bakcharskaya’, ‘Karina’, ‘Kuvshinovidnaya’, ‘Goluboe Vereteno’, and ‘Wojtek’. The average content of LAp was 31.72 mg/100 g fw. 7-*epi*-LAp appeared in all cultivars, and the average amount was 11.70 mg/100 g fw. ‘Bakcharskaya’, Atut’, and ‘Viola’ contained the lowest amount of this compound (2.86–3.69 mg/100 g fw), while ‘Morena’ contained the highest amount (20.43 mg/100 g fw). To our knowledge, there are no reports on the occurrence of pentose derivatives of loganic acid in other raw materials.

For all cultivars, the total content of Lo and S ranged from 1.87 mg/100 g fw (‘Tomichka’) to 71.71 mg/100 g fw (‘Wojtek’). The average content of these iridoids found in the berries was 19.00 mg/100 g fw, which accounted for 10% of all iridoids. These iridoids are not the main components of berries, but their presence in the fruit is important for their health benefits, such as antispasmodic and antibacterial activities [36,37]. The results of Ma et al. [38] showed that Lo and its derivatives were active against diabetic nephropathy. In addition, higher concentrations of L and S may increase the bitter taste of berries. Other fruits reported to contain both Lo and S are *Cornus officinalis* and *Cornus mas* [39,40,41]. According to Du et al. [39] and Zhou et al. [40], Lo, beside morroniside, is one of the main iridoids in the fruits of *C. officinalis*, while S is the minor iridoid in this fruit. Both compounds are present in trace amounts in *C. mas* fruits [41].

Among pentose derivatives of Lo, the most important compounds are Lop and 7-*epi*-Lop. Lop, similarly to LAp, was present in the same 24 cultivars. It was not detected in ‘Bakcharskaya’, ‘Karina’, ‘Kuvshinovidnaya’, ‘Goluboe Vereteno’, or ‘Wojtek’. Concentrations of Lp ranged from 5.85 mg/100 g fw in ‘Viola’ to 83.85 mg/100 g fw in ‘Amphora’. The average content of this compound was 35.79 mg/100 g fw, which accounted for 20% of all iridoids. 7-*epi*-Lop was found in only 14 cultivars. The lowest amount of this iridoid (1.83 mg/100 g fw) was found in ‘Chernichka’, whereas the highest (9.11 mg/100 g fw) was found in the ‘Blue Velvet’ cultivar. Similarly as in the case of the derivatives of LAp, there are no reports on the prevalence of the derivatives of Lop in other raw materials.

The 24 cultivars of berries contained secoLo. Among cultivars, the concentration of this compound ranged from 0.88 mg/100 g fw in ‘Leningradskii Velikan’ to 13.30 mg/100 g fw in ‘Klon 44’. secoLo occurs in *Lonicera* [42,43] and *Cornus* [44] species. According to Graikou et al. [37], secoLo, similarly to Lo, has antimicrobial activity but only against Gram-positive bacteria.

Table 3, Table 4, Table 5, Table 6 and Table 7 show the contents of individual phenolic compounds of 30 honeysuckle berry cultivars and genotypes. The major polyphenolic groups detected in honeysuckle berries in this study were similar to those reported in previous studies [3]. They were anthocyanins, flavonols, flavones, flavan-3-ols, and phenolic acids. Additionally, in this work, we identified compounds from another polyphenolic group, i.e., flavanonols.

Blue honeysuckle, like chokeberry and bilberry, is a rich source of anthocyanins [4,6,45]. The studied 30 cultivars of berries contain from 151.74 mg/100 g fw (‘Viola’) to 655.21 mg/100 g fw ('Amur') of these pigments (Table 3). Among the anthocyanins in honeysuckle berries, there were 5 compounds. Cy 3-glc was the major anthocyanin (83%–93%). High amounts of this anthocyanin were found in ‘Amur’ and ‘Klon 40’ (577.19 and 560.76 mg/100g), whereas low amounts were found in ‘Karina’ and ‘Viola’ (135.41 and 136.37 mg/100 g). The other four anthocyanins were present in smaller amounts: Cy 3-rut (1%–9%), Cy 3,5-diglc (2%–6%), Pn 3-glc (1%–5%), and Pn 3-glc and Pn 3-rut (>2%). Other authors, in addition to the above-mentioned six anthocyanins, have identified other compounds from this group, but their content is small [6,46].

Concentration of the three main flavonols (quercetin 3-*O*-rhamnoside-hexoside (Q-rha-hex), quercetin 3-*O*-vicianoside (Q-vic), and quercetin 3-*O*-glucoside (Q 3-glc)) in 30 honeysuckle berry cultivars and genotypes ranged from 10.65 mg/100 g fw (‘Roksana’) to 35.46 mg/100 g fw (‘Klon 40’) (Table 4). The dominant flavonol was Q-rha-hex. High amounts of this compound were found in ‘Amur’ and ‘Nympha’ (24.10 mg/100 g fw and 24.21 mg/100 g fw), whereas low amounts were observed in ‘Roksana’ (1.50 mg/100 g fw). The average content of Q-rha-hex, Q-vic, and Q 3-glc was 12.18 mg/100 g fw, 5.30 mg/100 g fw, and 4.13 mg/100 g fw, respectively. We previously identified the second of these compounds in chokeberry juices [47]. From the two identified flavanonols, only taxifolin 7-hexoside (Tx 7-hex) was quantitated (Table 4). The content of the compound ranged from 2.80 mg/100 g fw in ‘Viola’ to 14.56 mg/100 g fw in ‘Nympha’. A high concentration of this compound, above 10 mg/100 g fw, has also been observed in 5 cultivars of berries (‘Amur’, ‘Jolant’, ‘Kuvshinovidnaya’, ‘Morena’, and ‘Klon 40’). A lower content of derivatives of taxifolin was determined in fruits of *R. canina* and *R. micrantha* [31].

The concentration of flavones in the 30 cultivars and genotypes of honeysuckle berries was low. Among these compounds, only luteolin-trihexoside (L-trihex) was quantitatively determined; its content ranged from 0.09 mg/100 g (‘Klon 44’) to 1.07 mg/100 g fw (‘Morena’) (Table 4). It was not detected in ‘Bakcharskaya’ or ‘Roksana’. A similar level of L-trihex was observed in a previous study in ‘Wojtek’ cultivar [3]. According to Jurikova et al. [7], the content of luteolin 7-*O*-glucoside ranged from 4.70 mg to 13.60 mg in 100 g of berries of different *Lonicera* species. Flavones are rarely found in fruits. More often, they are present in vegetables, in particular in the leaves of celery [48].

The contents of (+)-catechin (Cat) and two procyanidin (PC) dimers in honeysuckle berries are shown in Table 5. Between tested cultivars, low levels of Cat and dimer 1 (*t*_R_ 2.66 min) were found in ‘Blue Velvet’, whereas ‘Dlinnoplodnaya’ contained high amounts. The content of dimer 2 (*t*_R_ 4.05 min) ranged from 0.40 mg/100 g fw (‘Wojtek’) to 20.92–21.77 mg/100 g fw (‘Viola’, ‘Chelyabinka’, ‘Vasilevskaya’, and ‘Roksana’).

The total content of quantified phenolic acids ranged from 27.11 mg/100 g fw in ‘Klon 44’ to 115.50 mg/100 g fw in ‘Tomichka’ (Table 6), and it was comparable to the quantities obtained by other authors [3,4]. High concentrations of these compounds have also been observed in 9 cultivars of berries (‘Bakcharskaya’, ‘Chelyabinka’, ‘Chernichka’, ‘Dlinnoplodnaya’, ‘Fialka’, ‘Kamchadalka’, ‘Sineglazka’, ‘Vasilevskaya’, and ‘Volshebnica’), with a value in the range 76.78–97.01 mg/100 g fw. 5-*O*-Caffeoylquinic acid (5-CQA, chlorogenic acid) was the most predominant phenolic acid found in berries and constituted on average more than 60% of the total amount of quantified hydroxycinnamic acids (HCA). The lowest amount of this compound (17.24 mg/100 g fw) was found in ‘Klon 44’, whereas the highest (60.37 mg/100 g fw) was found in the ‘Kamchadalka’ cultivar. The second acid present in large quantities (from 4.07 in ‘Nympha’ to 37.39 mg/100 g fw in ‘Tomichka’) was dicaffeoylquinic acid 1 (di-CQA 1). It constituted from 7% to 46% of the total amount of quantified phenolic acids. Similar results for these two acids have been reported by other authors [14,45]. The 3-*O*-caffeoylquinic acid (3-CQA) and caffeoylquinic acid (CQA) concentrations ranged from 0.35 mg/100 g fw (‘Amphora’) to 12.57 mg/100 g fw (‘Viola’), and from 0.16 mg/100 g fw (‘Kuwszinowidnaja’) to 8.97 mg/100 g fw (‘Chelyabinka’), respectively. The other two di-CQA isomers were present in smaller amounts: di-CQA 2 (0.05–3.21 mg/100 g fw) and di-CQA 3 (0.04–3.23 mg/100 g fw). Oszmiański et al. [3] for ‘Wojtek’ cv. reported the contents of di-CQA 2 and di-CQA 3 at the level of 0.15 mg/100 g fw, and this value was lower than the value for ‘Wojtek’ cv in this study. Other authors, among phenolic acids, identified only 3-CQA and 5-CQA, without the isomer di-CQA [14,45].

### 2.3. Antioxidant Activities of Cultivars and Ecotypes of Honeysuckle Berries

The antioxidant activity (1,1-Diphenyl-2-picrylhydrazyl radical (DPPH) and ferric reducing ability of plasma (FRAP)) of the blue honeysuckle berries of 30 cultivars and genotypes is shown in Table 7. The effects of compounds present in the berries on the antioxidant activity measured by the DPPH assay ranged from 8.80 to 28.77 μmol TE/g fw, and for the FRAP assay they ranged from 22.41 to 57.52 μmol TE/g fw. ‘Amur’, ‘Jolanta’, ‘Klon 40’, ‘Kuvshinovidnaya’, and ‘Nympha’ were characterized by high activity measured by both DPPH (26.48–28.77 μmol TE/g fw) and FRAP (50.24–57.52 μmol TE/g fw). Low activity was observed for ‘Atut’ (8.80 μmol TE/g fw for DPPH and 22.86 μmol TE/g fw for FRAP) and ‘Karina’ (8.94 μmol TE/g fw for DPPH and 22.41 μmol TE/g fw for FRAP). These results showed that DPPH and FRAP assays for 30 cultivars and genotypes revealed the same trend. Similar results for ABTS and FRAP assays have been reported previously by other authors [11]. High activity of honeysuckle berries has been confirmed by many authors [2,8,10,13], although the level of activity depends on many factors including cultivar. The differences in antioxidant activity between honeysuckle berry cultivars and genotypes may result from different qualitative and quantitative constituents.

The correlation between the antioxidant activity of honeysuckle berry extracts and the content of anthocyanins depends on the measurement method (Table S2). Correlation coefficients were higher for the FRAP method (*r* = 0.94) than the DPPH method (*r* = 0.78). A very strong correlation was observed between the antioxidant potential (FRAP) and anthocyanins (*r* = 0.94), and there was a strong correlation between the antioxidant potential (DPPH) and anthocyanins (*r* = 0.78).

These are the first studies on the correlation of the antioxidant activity of honeysuckle extract with the amount of iridoids. The obtained results showed that the amount of iridoids weakly correlated with in vitro antioxidant activity measured by DPPH and FRAP methods. This is due to the structure of iridoids, which in their molecule do not have (or they have, but only few) phenolic OH groups, which neutralize free radicals. This is consistent with the study of Pacifico et al. [49]. The authors reported that iridoids did not exhibit a good radical scavenging capacity. They explained that the moderate radical scavenging capacity is probably due to their poor hydrogen-donating ability. Iridoids exhibit higher biological activity, especially anti-inflammatory and antibacterial activity [33,34,35]; therefore their high content in blue honeysuckle berries can complement the antioxidant effect of phenolic compounds. Thus, honeysuckle berries may have wider potential than other fruits containing only polyphenols.

## 3. Materials and Methods

### 3.1. Reagent and Standard

1,1-Diphenyl-2-picrylhydrazyl radical (DPPH); 6-hydroxy-2,5,7,8-tetramethylchroman-2-carboxylic acid (Trolox); 2,4,6-tri(2-pyridyl)-s-triazine (TPTZ), dimethyl sulfoxide (DMSO), FeCl_3_, acetonitrile, formic acid, sweroside (S), and secologanin (SLo) were acquired from Sigma-Aldrich (Steinheim, Germany). Acetic acid was obtained from Chempur (Piekary Śląskie, Poland). Acetonitrile for LC–MS was purchased from POCh (Gliwice, Poland). Cyanidin 3-*O*-glucoside (Cy 3-glc) and p-coumaric acid (*p*-CuA), caffeic acid (CA), ferulic acid (FA), quercetin 3-*O*-glucoside, luteolin 7-*O*-glucoside (Lglc), luteolin 7-*O*-rutinoside, diosmin, hesperidin, naringin, rutin, (+)-catechin, (−)-epicatechin, procyanidin B1, loganic acid (LA), and loganin (Lo) were purchased from Extrasynthese (Lyon Nord, France). 5-*O*-caffeoylquinic acid (5-CQA, chlorogenic acid), 3-*O*-caffeoylquinic acid (3-CQA, neochlorogenic acid), and 4-*O*-caffeoylquinic acid (4-CQA, cryptochlorogenic acid) were purchased from TRANS MIT GmbH (Giessen, Germany). All reagents were of analytical grade.

### 3.2. Plant Materials

Honeysuckle berries (*Lonicera caerulea* L. var. *kamtschatica* Sevast.) from 6 cultivars (‘Amphora’, ‘Amur’, ‘Berry Smart Blue’, ‘Fialka’, ‘Morena’, ‘Nympha’) were harvested in the Arboretum and Institute of Physiography in Bolestraszyce (59°51′ N, 22°51′ E)), near Przemyśl, Poland, 14 cultivars (‘Atut’, ‘Bakczarskaja’, ‘Czelabinka’, ‘Czerniczka’, ‘Dilnnopłodnaja’, ‘Kamczadałka’, ‘Karina’, ‘Niebieskie Czeretenko’, ‘Roksana’, ‘Sinigłaska’, ‘Tomiczka’, ‘Viola’, ‘Wasilewkaja’, ‘Wołoszebnica’) and 2 genotypes (Klon 38 and 44) were harvested in the Research Station for Cultivar Testing in Masłowice (51°15′ N, 18°38′ E), Poland, 6 cultivars (‘Blue Velvet’, ‘Jolanta’, ‘Krupnopłodnaja’, ‘Kuwszinowidnaja’, ‘Leningradzkij Wielikan’, ‘Wojtek’) and 1 genotype (Klon 40) were harvested in the Polish Academy of Sciences Botanical Garden, Centre for Biological Diversity Conservation in Powsin (52°07′ N, 21°06′ E), and 1 cultivar (‘Vostorg’) was harvested in a horticultural farm in Dąbie near Silnowo (53°66′ N, 16°45′ E).

The plant materials were authenticated by Professor Jakub Dolatowski (Arboretum and Institute of Physiography in Bolestraszyce, Poland) and Aleksandra Siwik M.Sc. (the Research Station for Cultivar Testing in Masłowice).

### 3.3. Extraction of Compounds for Qualitative and Quantitative Analysis

Frozen berries of honeysuckle were homogenized and 5 g of the homogenate was extracted with 50 mL of 80% aqueous methanol (*v*/*v*) acidified with 1% HCl by ultrasonication for 20 min. The extract was centrifuged and diluted (re-distilled water with the ratio 1:1, *v*/*v*). For UPLC-qTOF-MS/MS and HPLC-PDA analysis the supernatant was filtered through a Hydrophilic PTFE 0.22 and 0.45 μm membrane (Millex Samplicity Filter, Merck, Germany) and used for routine investigation.

### 3.4. Identification of Iridoids and Polyphenols by UPLC-qTOF-MS/MS

The method was previously described by Wyspiańska et al. [50]. Identification of compounds was performed using the Acquity ultra-performance liquid chromatography (UPLC) system, coupled with a quadrupole-time of flight (Q-TOF) MS instrument (UPLC/Synapt Q-TOF MS, Waters Corp., Milford, MA, USA), with an electrospray ionization (ESI) source. Separation was achieved on an Acquity BEH C18 column (100 mm × 2.1 mm i.d., 1.7 μm; Waters). The mobile phase was a mixture of 2.0% aq. formic acid *v*/*v* (A) and acetonitrile (B). The gradient program was as follows: initial conditions—1% B in A, 12 min—25% B in A, 12.5 min—100% B, 13.5 min—1% B in A. The flow rate was 0.45 mL/min and the injection volume was 5 μL. The column was operated at 30 °C. UV–vis absorption spectra were recorded on-line during UPLC analysis, and the spectral measurements were made in the wavelength range of 200–600 nm, in steps of 2 nm. The major operating parameters for the Q-TOF MS were set as follows: capillary voltage 2.0 kV, cone voltage 40 V, cone gas flow of 11 L/h, collision energy 28–30 eV, source temperature 100 °C, desolvation temperature 250 °C, collision gas, argon; desolvation gas (nitrogen) flow rate, 600 L/h; data acquisition range, *m*/*z* 100–2000 Da; ionization mode, negative and positive. The data were collected with Mass-LynxTM V 4.1 software (Waters Corp., Milford, MA, USA). The runs were monitored at the following wavelengths: iridoids at 245 nm, phenolic acids and their derivatives at 320 nm, flavan-3-ols, flavonols, flavanonols, flavones and flavanones at 280 and 360 nm, anthocyanins at 520 nm.

### 3.5. Quantification of Iridoids and Polyphenols by HPLC-PDA

The analysis was previously described by Sokół-Łętowska et al. [51]. The HPLC-PDA analysis was performed using a Dionex (Germering, Germany) system equipped with the diode array detector model Ultimate 3000, quaternary pump LPG-3400A, autosampler EWPS-3000SI, thermostated column compartment TCC-3000SD, and controlled by Chromeleon v.6.8 software (Thermo Scientific Dionex, Sunnyvale, CA, USA). The Cadenza Imtakt column C5-C18 (75 × 4.6 mm, 5 μm) was used. The mobile phase was composed of solvent C (4.5% aq. formic acid, *v*/*v*) and solvent D (100% acetonitrile). The elution system was as follows: 0–1 min 5% D in C, 20 min 25% D in C, 21 min 100% D, 26 min 100% D, 27 min 5% D in C. The flow rate of the mobile phase was 1.0 mL/min and the injection volume was 20 μL. The column was operated at 30 °C. Iridoids were detected at 245 nm, flavan-3-ols at 280 nm, phenolic acids and their derivatives at 320 nm, flavonols, flavanonols, flavones and flavanones at 280 and 360 nm, and anthocyanins at 520 nm.

Loganic acid and its derivatives were expressed as mg of loganic acid equivalents (LAE) per 100 g fresh weight (fw), loganin, sweroside and their derivatives as loganin equivalents (LoE) per 100 g fw, anthocyanins as cyanidin 3-*O*-glucoside equivalents (CygE) per 100 g fw, derivatives of quercetin and taxifolin as quercetin 3-*O*-glucoside equivalents (QgE) per 100 g fw, luteolin -*O*-dihexoside-hexoside as luteolin 7-*O*-glucoside equivalents (LgE) per 100 g fw, caffeoylquinic acids as mg of 5-*O*-caffeoylquinic (chlorogenic) acid equivalents (ChAE) per 100 g fw. Solutions of standards (1 mg/ml) were dissolved in 1 mL of methanol. The appropriate amounts of stock solutions were diluted with 50% aqueous methanol (*v*/*v*) acidified with 1% HCl in order to obtain standard solutions. Analytical characteristics for determination of phenolic compounds and iridoids are shown in Table S3.

### 3.6. Antioxidant Capacity

The total antioxidant potential of samples was determined using a ferric reducing antioxidant power ability of plasma (FRAP) assay by Benzie and Strain [52] as a measure of antioxidant power. The FRAP reagent was prepared by mixing acetate buffer (300 μM, pH 3.6), a solution of 10 μM TPTZ in 40 μM HCl, and 20 μM FeCl_3_ at 10:1:1 (*v*/*v*/*v*). The DPPH radical scavenging activity of samples was determined according to the method of Yen and Chen [53]. DPPH (100 μM) was dissolved in pure ethanol (96%). All determinations were performed in triplicate using a UV-2401 PC spectrophotometer (Shimadzu, Kyoto, Japan). The absorbance was measured after 10 min at 593 nm for FRAP and at 517 nm for DPPH. For all analyses, a standard curve was prepared using different concentrations of Trolox. Calibration curves, in the range 0.01–5.00 μmol Trolox L^−1^, were used for the quantification of the three methods of antioxidant activity, showing good linearity (*r*^2^ ≥ 0.998). The results were corrected for dilution and expressed in μmol Trolox equivalent (TE) per 100 g fw.

### 3.7. Statistical Analyses

Results were presented as the mean ± standard deviation of three technical replications. All statistical analyses were performed with Statistica version 12.0 (StatSoft, Tulsa, OK, USA). One-way analysis of variance (ANOVA) by Duncan’s test was used to compare the mean values. Differences were considered to be significant at α = 0.05.

## 4. Conclusions

The reported research clearly shows that honeysuckle berries are an excellent source not only of polyphenols (mainly anthocyanins), but also of iridoids, which are rarely present in other fruits. The content of both iridoids and polyphenols and antioxidant activity measured by DPPH and FRAP methods depends largely on the cultivar of berries. All 27 cultivars and 3 genotypes of blue honeysuckle berries had similar anthocyanin, flavonol, flavanonol, flavone, flavan-3-ol, and phenolic acid profiles, but did not have similar iridoid profiles. Two iridoids, two flavanones, and three flavones were identified in honeysuckle berries for the first time. The content of iridoids, besides anthocyanins, may be one of the most important parameters for appraising the characterization of blue honeysuckle fruits with respect to their nutraceutical value and potential use for different purposes.

## Figures and Tables

**Table 1:** Characterization of compounds of honeysuckle fruits determined using their spectral characteristics in positive and negative ions in ultra-performance liquid chromatography coupled with electrospray ionization mass spectrometry (UPLC-ESI-qTOF-MS/MS).

Peak No.	*t*_R_ (min)	UV λ_max_ (nm)		[M − H]^−^/[M + H]^+^ (*m*/*z*)	Other Ions (*m*/*z*)	Compound
1	2.45	245, 325		353.0879	191.0553	3-*O*-caffeoylquinic acid
2	2.66	242, 278		577.1349	289.0723/287.0540	procyanidin dimer
3	2.80	245		375.1276	213.0769/191.0553/151.0771/125.0585	8-*epi*-loganic acid
4	2.98	243, 332		629.1733 ^+^	467.1172/449.1063/305.0649/287.0536/137.0236	taxifolin 7-*O*-dihexoside
5	3.10	242, 278		865.1967	577.1349/289.0723/287.0540	procyanidin trimer
6	3.22	242, 278		289.0723	245.0787	(+)-catechin
7	3.49	245, 325		341.0849	179.0349/135.0440	caffeoylglucose
8	3.62	245, 325		353.0879	191.0553	5-*O*-caffeoylquinic acid
9	3.73	245		375.1276	213.0769/191.0553/169.0855/151.0771/119.0331	loganic acid
10	3.89	278, 513		611.1664 ^+^	449.1063/287.0536	cyanidin 3,5-*O*-diglucoside
11	4.05	242, 278		577.1349	289.0723	procyanidin dimer
12	4.17	245		507.1746	375.1356/213.0769/169.0855	pentosyl-loganic acid
13	4.27	245		375.1276	213.0769/195.0678/151.0771/121.0648/119.0331	7-*epi*-loganic acid
14	4.38	243, 340		467.1172 ^+^	449.1063/305.0649/287.0536/137.0236	taxifolin 7-*O*-hexoside
15	4.49	245		771.2018	609.1432/447.0959/285.0394	luteolin *O*-trihexoside
16	4.53	245		507.1746	375.1356/345.0806/213.0769/169.0855/151.0746	loganic acid 7-*O*-pentoside
17	4.74	280, 514		449.1107 ^+^	287.0536	cyanidin 3-*O*-glucoside
18	5.02	325		353.0880	191.0553	caffeoylquinic acid
19	5.12	245		507.1700	357.1144/327.1092/195.0650/151.0771	7-*epi*-loganic acid 7-*O*-pentoside
20	5.22	242, 278		865.1967	577.1349/289.0723	procyanidin trimer
21	5.22	280, 517		595.1664 ^+^	287.0536	cyanidin 3-*O*-rutinoside
22	5.50	242, 278		1153.2568	865.1967/577.1349/289.0723	procyanidin tetramer
23	5.61	280, 500		433.1125 ^+^	271.0601	pelargonidin 3-*O*-glucoside
24	5.68	245		521.1854	389.1423/227.0908 (567.1918 [M − H + HCOOH]^−^)	pentosyl-loganin
25	5.73	245		357.1183	195.0650/125.0241 (403.1223 [M − H + HCOOH]^−^)	sweroside
26	5.73	245		389.1342	227.0939/209.0805 (435.1502 [M − H + HCOOH]^−^)	loganin
27	5.93	245		489.1630	195.0650/125.0241 (535.1502 [M − H + HCOOH]^−^)	pentosyl-sweroside
28	6.10	279, 516		463.1247 ^+^	301.1730	peonidin 3-*O*-glucoside
29	6.15	245		521.1854	389.1342/227.0939 (567.1918 [M − H + HCOOH]^−^)	loganin 7-*O*-pentoside
30	6.34	352		625.1386	301.0354	quercetin *O*-dihexoside
31	6.36	278, 519		609.1807 ^+^	301.0730	peonidin 3-*O*-rutinoside
32	6.54		245	389.1342	227.0939/209.0805(435.1502 [M − H + HCOOH]^−^)	7-*epi*-loganin
33	6.58		245	403.1263	223.0622/165.0565/121.0288	secoxyloganin
34	6.74		353	595.1312	301.0354	quercetin *O*-vicianoside ^1^ 1
35	6.78		245	489.1630	389.1342/371.0636/227.0939/209.0805 (567.1918 [M − H + HCOOH]^−^)	7-*epi*-loganin 7-*O*-pentoside
36	6.87		353	595.1312	301.0354	quercetin *O*-vicianoside 2
37	6.89		245	433.1331	225.0781/155.0347 (433.1331 [M − H + HCOOH]^−^)	secologanin
38	7.07		352	609.1483	301.0354	quercetin 3-*O*-rutinoside
39	7.32		352	609.1483	463.0887/301.0354	quercetin *O*-rhamnoside-*O*-hexoside
40	7.49		352	463.0887	301.0354	quercetin 3-*O*-glucoside
41	7.58		346	593.1507	285.0394	luteolin 7-*O*-rutinoside
42	7.68		346	447.0918	285.0394	luteolin 7-*O*-glucoside
43	7.80		352	433.0777	301.0354	quercetin *O*-pentoside
44	8.01		346	593.1507	447.0918/285.0394	luteolin 7-*O*-deoxyhexosyl-*O*-hexoside
45	8.20		245, 327	609.1483	447.0916/315.0512	isorhamnetin-*O*-hexosyl-*O*-pentoside
46	8.39		326	515.1196	353.0879/191.0553	dicaffeoylquinic acid 1
47	8.70		325	515.1196	353.0879/191.0553	dicaffeoylquinic acid 2
48	8.92		352	505.0979	301.0319	quercetin *O*-acetyl-hexoside
49	9.38		326	515.1196	353.0879/191.0553	dicaffeoylquinic acid 3
50	9.54		339	607.1697	299.0536/284.0382	diosmetin 7-*O*-rutinoside

^+^ positive electrospray ionisation; ^1^ vicianoside, 6-*O*-α-l-arabinosyl-d-glucose.

**Table 2:** Iridoid content (mg/100 g fw) of different blue honeysuckle cultivars and genotypes.

Cultivar/genotype	LA	7-*epi*-LA	LAp	7-*epi*-LAp	S+Lo	Lop	7-*epi*-Lop	secoLo	Total Ir
‘Amphora’	60.44 ± 1.07no^1^		44.74 ± 0.44e	12.22 ± 0.90f	25.82 ± 2.10g	83.85 ± 1.02a		4.91 ± 0.34gh	231.98d
‘Amur’	136.85 ± 4.12c		36.67 ± 1.11g	9.78 ± 0.23gh	19.64 ± 3.06i	45.83 ± 2.34e	3.52 ± 0.06c	7.61 ± 0.37e	259.89b
‘Atut’	63.29 ± 1.24n		16.71 ± 0.52m	3.35 ± 0.07k	22.04 ± 2.15hi	70.29 ± 1.49b	6.62 ± 0.05b	11.84 ± 0.26bc	194.13h
‘Bakcharskaya Y.’ ^1^	90.18 ± 2.20i			2.86 ± 0.30k	50.01 ± 2.62c		3.51 ± 0.19c	6.20 ± 0.18f	152.77kl
‘Berry Smart Blue’	126.48 ± 0.48e	21.63 ± 0.14d	31.22 ± 0.88h	17.29 ± 0.55cd	24.56 ± 0.79gh	40.74 ± 0.52f	2.75 ± 0.31de	11.77 ± 0.06bc	276.43a
‘Blue Velvet’	51.12 ± 2.71r		9.37 ± 0.16o	15.03 ± 1.62e	7.34 ± 0.15lmn	47.27 ± 0.52de	9.11 ± 0.35a	5.05 ± 0.12gh	144.29mn
‘Chelyabinka’	39.21 ± 0.50wz	23.36 ± 0.77c	19.25 ± 0.60l	6.18 ± 0.09j	4.22 ± 0.16nop	32.43 ± 1.55gh			124.67o
‘Chernichka’	57.70 ± 0.81op	2.45 ± 0.13g	28.28 ± 0.15i	13.50 ± 0.88ef	3.80 ± 0.50op	12.11 ± 0.21m	1.83 ± 0.11f	3.77 ± 0.14i	123.46o
‘Dlinnoplodnaya’	41.44 ± 0.69tw	1.79 ± 0.09g	37.39 ± 0.17g	10.64 ± 0.03g	3.46 ± 0.23op	25.24 ± 0.59k			119.95o
‘Fialka’	56.52 ± 0.11op		13.23 ± 1.10n	14.13 ± 0.23e	40.94 ± 4.45e	70.34 ± 0.38b	2.32 ± 0.30def	11.20 ± 0.91c	208.71f
‘Goluboe Vereteno’	118.46 ± 3.52f	26.28 ± 0.38b		13.81 ± 0.34e	32.83 ± 1.62f		2.60 ± 0.14de	3.49 ± 0.96i	197.47gh
‘Jolanta’	55.11 ± 1.12pr	10.37 ± 1.06e	28.94 ± 0.13i	9.59 ± 0.00gh	4.12 ± 0.02nop	25.75 ± 0.11k		5.24 ± 0.05g	139.13n
‘Kamchadalka’	67.74 ± 2.57m		71.16 ± 0.48a	10.13 ± 0.85gh	6.48 ± 0.37mno	33.81 ± 0.41g	2.15 ± 0.11ef	12.15 ± 0.34b	203.61fg
‘Karina’	116.17 ± 1.17f			18.44 ± 0.42bc	68.27 ± 2.16b		3.68 ± 0.07c	9.55 ± 0.11d	216.11e
‘Klon 38’	46.64 ± 1.17s	10.54 ± 0.22e	22.63 ± 0.46k	8.58 ± 0.66hi	3.57 ± 0.49op	26.70 ± 0.69jk		4.87 ± 0.09gh	123.53o
‘Klon 40’	76.72 ± 1.39k	8.48 ± 1.44f	61.88 ± 1.74b	10.71 ± 0.74g	6.64 ± 0.23mno	40.08 ± 0.10f		0.88 ± 0.04j	205.40f
‘Klon 44’	69.76 ± 0.83m		13.72 ± 1.92n	9.42 ± 0.01gh	12.54 ± 0.30jk	49.44 ± 2.06d	3.81 ± 0.04c	13.30 ± 0.57a	171.98i
‘Krupnoplodnaya’	106.94 ± 0.18g		22.62 ± 1.08k	17.47 ± 0.34cd	4.76 ± 0.61nop	25.58 ± 0.36k		1.30 ± 0.28j	178.67i
‘Kuvshinovidnaya’	182.28 ± 2.64a			19.26 ± 0.47ab	45.68 ± 0.31d				247.21c
‘Leningradskii V.’	131.69 ± 0.84d		47.40 ± 0.13d	15.01 ± 0.72e	3.36 ± 0.64op	19.22 ± 0.76l		0.88 ± 0.01j	217.57e
‘Morena’	161.36 ± 5.85b		35.76 ± 0.36g	20.43 ± 1.46a	8.75 ± 0.10lm	38.87 ± 2.68f		7.47 ± 0.96e	272.63a
‘Nympha’	71.35 ± 1.68lm		26.23 ± 0.22j	9.75 ± 0.33gh	14.25 ± 2.81j	32.71 ± 0.42g		4.17 ± 0.19hi	158.46jk
‘Roksana’	58.15 ± 0.58op		49.49 ± 0.22c	6.75 ± 0.77j	10.56 ± 0.01kl	21.30 ± 0.25l		3.76 ± 0.59i	150.01lm
‘Sineglazka’	38.49 ± 0.51wz	24.12 ± 0.40c	19.45 ± 0.29l	6.68 ± 0.22j	4.91 ± 0.01nop	29.01 ± 0.39ij			122.66o
‘Tomichka’	84.92 ± 2.04j		20.09 ± 0.25l	7.36 ± 1.55ij	1.87 ± 0.32p	10.87 ± 0.12m			125.10o
‘Vasilevskaya’	74.04 ± 0.55kl		24.90 ± 1.14j	17.87 ± 0.66bcd	18.89 ± 0.72i	29.98 ± 0.02hi	2.80 ± 0.24d	9.42 ± 0.10d	177.90i
‘Viola’	45.48 ± 2.35st	45.05 ± 0.62a	40.57 ± 1.16f	3.69 ± 0.02k	9.61 ± 0.23klm	5.85 ± 0.85n			150.25lm
‘Volshebnica’	68.84 ± 0.65m		19.97 ± 0.80l	14.58 ± 0.44e	20.45 ± 0.94i	20.94 ± 0.43l	4.01 ± 0.63c	13.29 ± 0.01a	162.07j
‘Vostorg’	35.22 ± 2.35z		50.32 ± 1.81c	9.81 ± 0.67gh		56.59 ± 3.40c		9.30 ± 0.86	161.24j
‘Wojtek’	100.13 ± 1.19h			16.58 ± 0.84d	71.71 ± 0.18a		4.02 ± 0.29c	13.30 ± 0.06a	205.74f

LA, loganic acid; 7-*epi*-LA, 7-*epi* loganic acid; LAp, loganic acid 7-*O*-pentoside; 7-*epi*-LAp, 7-*epi*-loganic acid 7-*O*-pentoside; S, sweroside; Lo, loganin; Lop, loganin 7-*O*-pentoside; secoLo, secologanin; Total Ir, total amount of quantified iridoids; ^1^ ‘Bakcharskaya Y.’, ‘Bakcharskaya Yubileynaya’; ‘Leningradskii V.’, ‘Leningradskii Velikan’. ^1^ Values are expressed as the mean (n = 3) ± standard deviation. Mean values with different letters (a, b, c, etc.) within the same column are statistically different (*p* < 0.05).

**Table 3:** Anthocyanin content (mg/100 g fw) of different blue honeysuckle cultivars and genotypes.

Cultivar/Genotype	Cy 3,5-diglc	Cy 3-glc	Cy 3-rut	Pg 3-glc	Pn 3-glc	Pn 3-rut	Total A
‘Amphora’	27.42 ± 0.65b ^1^	400.11 ± 14.67de	27.68 ± 0.10a	2.18 ± 0.07e	12.25 ± 0.38g	1.69 ± 0.07de	471.32d
‘Amur’	32.10 ± 1.02a	577.19 ± 20.46a	14.38 ± 0.04g	9.02 ± 0.29a	21.26 ± 0.71b	1.25 ± 0.01f	655.21a
‘Atut’	11.57 ± 0.64j	163.19 ± 8.17no	1.83 ± 0.11rs	0.44 ± 0.05lmn	5.20 ± 0.27p	0.15 ± 0.03n	182.37o
‘Bakcharskaya Y.’ ^2^	5.24 ± 0.07pr	217.02 ± 2.57jkl	4.92 ± 0.09o	4.76 ± 0.01b	5.43 ± 0.10op	0.36 ± 0.00kl	237.73klm
‘Berry Smart Blue’	11.28 ± 0.42j	239.44 ± 15.97hi	24.66 ± 0.29c	1.02 ± 0.05i	10.39 ± 0.63h	1.85 ± 0.06c	288.63hi
‘Blue Velvet’	11.29 ± 0.14j	216.04 ± 1.79jkl	2.65 ± 0.06pr	0.47 ± 0.02lm	5.52 ± 0.02op	0.30 ± 0.01lm	236.26klm
‘Chelyabinka’	16.59 ± 0.21fg	280.73 ± 2.56g	6.44 ± 0.14klm	1.32 ± 0.05g	9.84 ± 0.11hi	0.55 ± 0.01j	315.46fg
‘Chernichka’	6.43 ± 0.01no	179.66 ± 1.15mn	6.82 ± 0.03k	0.39 ± 0.03mn	5.91 ± 0.04nop	0.43 ± 0.00k	199.64no
‘Dlinnoplodnaya’	15.94 ± 0.11g	290.68 ± 2.60g	14.51 ± 0.10g	3.52 ± 0.05c	8.36 ± 0.02kl	0.63 ± 0.01ij	333.65f
‘Fialka’	18.24 ± 0.11e	387.53 ± 0.47ef	9.82 ± 1.60ij	1.28 ± 0.07g	14.54 ± 0.05e	1.12 ± 0.02g	432.54e
‘Goluboe Vereteno’	9.43 ± 0.12k	245.44 ± 10.20gh	7.26 ± 0.13k	0.58 ± 0.04kl	10.47 ± 0.35h	0.64 ± 0.02ij	273.82ij
‘Jolanta’	20.40 ± 0.52d	511.40 ± 25.23b	11.74 ± 0.92h	2.18 ± 0.10e	12.98 ± 0.51f	0.30 ± 0.00lm	559.02c
‘Kamchadalka’	3.64 ± 0.10s	204.41 ± 5.73jkl	10.27 ± 0.09i	0.21 ± 0.01o	5.50 ± 0.14op	0.58 ± 0.01j	224.61lm
‘Karina’	5.20 ± 0.08pr	135.41 ± 2.76p	7.36 ± 0.32k	0.30 ± 0.00no	1.77 ± 0.10t	0.27 ± 0.00m	150.31p
‘Klon 38’	6.57 ± 0.05n	234.71 ± 8.75hi	5.18 ± 0.17no	0.80 ± 0.02j	5.55 ± 0.25op	0.32 ± 0.00lm	253.13jk
‘Klon 40’	20.94 ± 0.17d	560.76 ± 6.58a	14.82 ± 0.02g	2.57 ± 0.01d	14.45 ± 0.21e	1.19 ± 0.03fg	614.72b
‘Klon 44’	14.88 ± 0.69h	222.40 ± 19.41ij	1.94 ± 0.13rs	0.70 ± 0.01jk	8.47 ± 0.68jkl	0.16 ± 0.02n	248.54kl
‘Krupnoplodnaya’	3.49 ± 0.07s	155.85 ± 2.34op	5.89 ± 0.03lmn	0.47 ± 0.02lm	9.16 ± 0.15ij	0.74 ± 0.00h	175.60o
‘Kuvshinovidnaya’	14.89 ± 0.53h	422.28 ± 15.91c	17.60 ± 0.66e	1.09 ± 0.05hi	23.35 ± 0.85a	2.09 ± 0.05b	481.30d
‘Leningradskii V.’	5.61 ± 0.08p	197.88 ± 0.31lm	6.70 ± 0.13kl	0.74 ± 0.02j	8.64 ± 0.02jk	0.70 ± 0.01hi	220.27mn
‘Morena’	22.27 ± 0.37c	409.92 ± 1.41cd	22.34 ± 0.03d	3.48 ± 0.02c	14.08 ± 0.06e	1.62 ± 0.14e	473.71d
‘Nympha’	13.03 ± 0.03i	496.30 ± 0.05b	26.65 ± 0.14b	1.49 ± 0.00f	18.46 ± 0.07c	1.74 ± 0.07d	557.68c
‘Roksana’	5.68 ± 0.12p	181.93 ± 3.68mn	3.23 ± 0.11p	0.22 ± 0.01o	5.99 ± 0.19no	0.25 ± 0.02m	197.31o
‘Sineglazka’	16.93 ± 0.13f	272.89 ± 1.47g	5.64 ± 0.17mno	1.10 ± 0.00hi	9.21 ± 0.05ij	0.57 ± 0.03j	306.33gh
‘Tomichka’	4.54 ± 0.07pr	175.66 ± 1.67no	9.22 ± 0.09j	1.23 ± 0.02gh	4.40 ± 0.06r	0.43 ± 0.00k	195.49o
‘Vasilevskaya’	7.38 ± 0.10m	200.04 ± 0.72klm	6.88 ± 0.15k	2.05 ± 0.01e	7.89 ± 0.01lm	0.55 ± 0.02j	224.79lm
‘Viola’	5.81 ± 0.10op	136.37 ± 4.42p	1.47 ± 0.03s	1.46 ± 0.09f	6.50 ± 0.20n	0.13 ± 0.00n	151.74p
‘Volshebnica’	8.02 ± 0.10lm	221.09 ± 4.05ijk	5.90 ± 0.06lmn	0.36 ± 0.01mno	7.58 ± 0.16m	0.62 ± 0.01ij	243.57klm
‘Vostorg’	8.63 ± 0.40l	368.23 ± 13.66f	27.94 ± 1.16a	0.55 ± 0.03l	15.30 ± 0.64d	2.22 ± 0.09a	422.87e
‘Wojtek’	10.90 ± 0.02j	251.00 ± 4.83h	15.67 ± 0.08f	0.81 ± 0.00j	2.98 ± 0.04s	0.30 ± 0.01m	281.66i

Cy 3,5-diglc, cyanidin 3,5-*O*-diglucoside; Cy 3-glc, cyanidin 3-*O*-glucoside; Cy 3-rut, cyanidin 3-*O*-rutinoside; Pg 3-glc, pelargonidin 3-*O*-glucoside; Pn 3-glc, peonidin 3-*O*-glucoside; Pn 3-rut, peonidin 3-*O*-rutinoside; Total A, total amount of quantified anthocyanins; ^1^ Values are expressed as the mean (n = 3) ± standard deviation. Mean values with different letters (a, b, c, etc.) within the same column are statistically different (*p* < 0.05). ^2^ ‘Bakcharskaya Y.’, ‘Bakcharskaya Yubileynaya’; ‘Leningradskii V.’, ‘Leningradskii Velikan’.

**Table 4:** Flavonol, flavanonol, and flavanone contents (mg/100 g fw) of different blue honeysuckle cultivars and genotypes.

Cultivar/Genotype	Q-vic	Q-rha-hex	Q 3-glc	Total Q	Tx 7-hex	L-trihex
‘Amphora’	1.54 ± 0.10p^1^	14.44 ± 0.42g	6.42 ± 0.10e	22.40g	8.57 ± 0.22f	0.80 ± 0.05c
‘Amur’	2.76 ± 0.14n	24.10 ± 0.63a	7.06 ± 0.63d	33.92b	13.83 ± 0.42b	0.54 ± 0.01e
‘Atut’	4.26 ± 0.21kl	14.12 ± 0.67gh	7.88 ± 0.33c	26.27e	4.06 ± 0.22lm	0.26 ± 0.02jkl
‘Bakcharskaya Y.’ ^2^	8.60 ± 0.51e	2.44 ± 0.15t	3.18 ± 0.19lm	14.22kl	6.64 ± 0.10gh	
‘Berry Smart Blue’	1.03 ± 0.04r	13.66 ± 0.33hi	1.60 ± 0.04p	16.29ij	6.36 ± 0.36ghi	0.25 ± 0.01jkl
‘Blue Velvet’	3.29 ± 0.04m	5.08 ± 0.01r	4.19 ± 0.05ijk	12.56m	6.94 ± 0.23g	0.19 ± 0.01klm
‘Chelyabinka’	7.94 ± 0.03f	12.65 ± 0.28j	3.81 ± 0.09k	24.39f	6.31 ± 0.06ghi	0.26 ± 0.00jkl
‘Chernichka’	1.77 ± 0.13op	12.39 ± 0.33jk	1.40 ± 0.09p	15.55ij	4.74 ± 0.15kl	0.30 ± 0.00ij
‘Dlinnoplodnaya’	2.56 ± 0.12n	17.81 ± 0.19d	2.06 ± 0.05o	22.44g	6.62 ± 0.16gh	0.30 ± 0.02ij
‘Fialka’	1.91 ± 0.02o	15.75 ± 0.06f	4.33 ± 0.21ij	21.99g	9.78 ± 0.11e	0.43 ± 0.01fg
‘Goluboe Vereteno’	1.79 ± 0.05op	11.48 ± 0.29l	3.15 ± 0.33lm	16.42i	6.38 ± 0.45ghi	0.35 ± 0.02hi
‘Jolanta’	8.31 ± 0.15e	12.43 ± 0.19jk	8.36 ± 0.26b	29.10d	11.83 ± 0.05c	0.32 ± 0.10hij
‘Kamchadalka’	10.19 ± 0.24ab	7.98 ± 0.15o	4.00 ± 0.13jk	22.17g	5.93 ± 0.02hij	0.26 ± 0.01jk
‘Karina’	1.04 ± 0.02r	9.55 ± 0.25n	1.41 ± 0.04p	12.00m	3.22 ± 0.07no	0.38 ± 0.00gh
‘Klon 38’	9.89 ± 0.04bc	13.31 ± 0.19i	6.04 ± 0.13e	29.24d	5.90 ± 0.15hij	0.11 ± 0.01n
‘Klon 40’	10.40 ± 0.16a	15.97 ± 0.33f	9.09 ± 0.10a	35.46a	11.32 ± 0.60cd	0.38 ± 0.01gh
‘Klon 44’	4.60 ± 0.26j	4.02 ± 0.28s	5.47 ± 0.40f	14.10kl	5.78 ± 0.38ij	0.09 ± 0.01n
‘Krupnoplodnaya’	6.59 ± 0.05h	6.38 ± 0.04p	2.76 ± 0.07mn	15.73ij	4.44 ± 0.10l	0.12 ± 0.01n
‘Kuvshinovidnaya’	3.95 ± 0.07l	11.91 ± 0.30kl	4.58 ± 0.09hi	20.44h	11.02 ± 0.62d	0.95 ± 0.05b
‘Leningradskii V.’	7.74 ± 0.03f	8.30 ± 0.01o	4.20 ± 0.09ijk	20.25h	5.84 ± 0.19hij	0.37 ± 0.01gh
‘Morena‘	7.27 ± 0.02g	18.92 ± 0.32c	4.15 ± 0.01ijk	30.34d	11.25 ± 0.73cd	1.07 ± 0.02a
‘Nympha’	2.60 ± 0.07n	24.21 ± 0.03a	4.97 ± 0.22gh	31.79c	14.56 ± 0.28a	0.63 ± 0.03d
‘Roksana’	6.12 ± 0.13i	1.50 ± 0.03w	3.03 ± 0.12lmn	10.65n	5.33 ± 0.02jk	
‘Sineglazka’	6.81 ± 0.01h	10.29 ± 0.30m	3.36 ± 0.21l	20.47h	6.41 ± 0.06ghi	0.19±0.02m
‘Tomichka’	0.81 ± 0.01s	21.29 ± 0.08b	1.28 ± 0.10p	22.57g	3.70 ± 0.20mn	0.20±0.04klm
‘Vasilevskaya’	9.28 ± 0.05d	10.32 ± 0.10m	4.83 ± 0.10gh	24.43f	5.76 ± 0.09ij	0.46 ± 0.00f
‘Viola’	9.82 ± 0.22c	9.59 ± 0.21n	5.22 ± 0.09fg	24.62f	2.80 ± 0.10o	0.27 ± 0.01j
‘Volshebnica’	4.39 ± 0.05jk	7.99 ± 0.14o	2.65 ± 0.04n	15.03jk	5.78 ± 0.13ij	0.37 ± 0.02ghi
‘Vostorg’	6.03 ± 0.38i	16.97 ± 0.86e	2.05 ± 0.06o	25.47ef	5.88 ± 1.01nij	0.42 ± 0.02fg
‘Wojtek’	1.14 ± 0.03r	10.57 ± 0.05m	1.45 ± 0.04p	13.16lm	5.38 ± 0.06jk	0.63 ± 0.04d

Q-vic, quercetin *O*-vicianoside 1; Q-rha-hex, quercetin *O*-rhamnoside-*O*-hexoside; Q 3-glc, quercetin 3-*O*-glucoside; Total Q, total amount of quantified derivatives of quercetin; Tx 7-hex, taxifolin 3-O-hexoside; L-trihex, luteolin trihexoside; ^1^ Values are expressed as the mean (n = 3) ± standard deviation. Mean values with different letters (a, b, c, etc.) within the same column are statistically different (*p* < 0.05). ^2^ ‘Bakcharskaya Y.’, ‘Bakcharskaya Yubileynaya’; ‘Leningradskii V.’, ‘Leningradskii Velikan’.

**Table 5:** Flavan-3-ol derivatives content (mg/100 g fw) of different blue honeysuckle cultivars and genotypes.

Cultivar/Genotype	PC Dimer 1	(+)-Catechin	PC Dimer 2	Total F
‘Amphora’	7.93 ± 0.64h ^1^	10.52 ± 1.02h	7.80 ± 0.29fg	26.26ij
‘Amur’	11.91 ± 0.24d	12.46 ± 0.47fg	4.37 ± 0.07j	28.74h
‘Atut’	3.08 ± 0.45r	4.31 ± 0.29no	5.43 ± 0.48hi	12.82m
‘Bakcharskaya Y.’ ^2^	6.01 ± 0.28jklm	9.14 ± 0.02i	12.39 ± 0.33d	27.54hi
‘Berry Smart Blue’	11.37 ± 0.41de	17.15 ± 0.59d		28.52h
‘Blue Velvet’	0.89 ± 0.04s	2.14 ± 0.03r	1.55 ± 0.01l	4.58o
‘Chelyabinka’	14.87 ± 0.13b	17.22 ± 0.04d	20.95 ± 0.61a	53.04b
‘Chernichka’	5.57 ± 0.08lmn	7.11 ± 0.06j	12.33 ± 0.57d	25.00j
‘Dlinnoplodnaya’	18.38 ± 0.77a	31.22 ± 0.44a	5.76 ± 0.23h	55.36a
‘Fialka’	5.63 ± 1.19lmn	6.10 ± 0.58kl	9.37 ± 0.34e	21.10k
‘Goluboe Vereteno’	13.08 ± 0.10c	23.05 ± 0.01b		36.12f
‘Jolanta’	12.04 ± 0.08d	4.23 ± 0.32no	4.62 ± 0.29ij	20.89k
‘Kamchadalka’	4.68 ± 0.19op	11.75 ± 0.27g	14.85 ± 0.36c	31.28g
‘Karina’	10.48 ± 0.02f	15.80 ± 0.56e	2.76 ± 0.10k	29.05h
‘Klon 38’	5.29 ± 0.08mno	6.61 ± 0.10jk	8.63 ± 0.09ef	20.53k
‘Klon 40’	9.58 ± 0.29g	5.48 ± 0.18lm	4.72 ± 0.11ij	19.77kl
‘Klon 44’	3.30 ± 0.47r	2.84 ± 0.36pr	7.38 ± 0.29g	13.52m
‘Krupnoplodnaya’	6.54 ± 0.10ijk	3.32 ± 0.03op	16.05 ± 0.02b	25.91ij
‘Kuvshinovidnaya’	3.27 ± 0.13r	2.04 ± 0.31r	3.90 ± 0.64j	9.21n
‘Leningradskii V.’	4.04 ± 0.08p	4.46 ± 0.10n	16.13 ± 0.07b	24.63j
‘Morena‘	5.86 ± 0.08klm	9.69 ± 0.33hi	9.01 ± 0.20e	24.56j
‘Nympha’	4.85 ± 0.07no	4.91 ± 0.37mn	8.58 ± 0.32ef	18.34l
‘Roksana’	6.80 ± 0.18ij	10.67 ± 0.90h	21.77 ± 1.09a	39.24e
‘Sineglazka’	14.91 ± 0.04b	17.71 ± 0.44d	15.10 ± 0.30c	47.72d
‘Tomichka’	10.88 ± 0.16ef	17.55 ± 0.65d		28.43h
‘Vasilevskaya’	9.34 ± 0.71g	19.85 ± 1.27c	21.53 ± 0.83a	50.72c
‘Viola’	1.62 ± 0.04s	10.02 ± 0.11hi	20.92 ± 0.08a	32.55g
‘Volshebnica’	6.13 ± 0.12ijkl	9.86 ± 0.04hi	12.66 ± 0.36d	28.66h
‘Vostorg’	6.85 ± 0.14i	12.76 ± 0.03f	8.96 ± 0.66e	28.57h
‘Wojtek’	2.94 ± 0.08r	9.32 ± 0.13i	0.40 ± 0.06m	12.65m

Total F, total amount of quantified flavan-3-ols; ^1^ Values are expressed as the mean (n = 3) ± standard deviation. Mean values with different letters (a, b, c, etc.) within the same column are statistically different (*p* < 0.05). ^2^ ‘Bakcharskaya Y.’, ‘Bakcharskaya Yubileynaya’; ‘Leningradskii V.’, ‘Leningradskii Velikan’.

**Table 6:** Phenolic acid content (mg/100 g fw) of different blue honeysuckle cultivars and genotypes.

Cultivar/Genotype	3-CQA	5-CQA	CQA	di CQA 1	di CQA 2	di CQA 3	Total PA
‘Amphora’	0.35 ± 0.00n ^1^	36.43 ± 0.91i	4.42 ± 0.35ij	5.44 ± 0.22no	1.04 ± 0.07efgh	0.34 ± 0.04jkl	48.01jk
‘Amur’	3.50 ± 0.13h	46.31 ± 1.35e	5.45 ± 0.18f	5.81 ± 0.31no	1.01 ± 0.01efgh	0.33 ± 0.02jklm	62.40ghi
‘Atut’	0.57 ± 0.04mn	25.22 ± 0.97l	4.11 ± 0.03k	11.86 ± 0.56ijkl	1.17 ± 0.06defg	0.95 ± 0.01ef	43.88kl
‘Bakcharskaya Y.’ ^2^	3.66 ± 0.05h	50.74 ± 0.10d	4.02 ± 0.01k	33.93 ± 3.23b	1.86 ± 0.16c	1.40 ± 0.16d	95.62b
‘Berry Smart Blue’	3.53 ± 0.05h	33.88 ± 0.05j	5.13 ± 0.22fg	5.92 ± 0.11no	0.66 ± 0.02fhi	0.50 ± 0.01ij	49.61j
‘Blue Velvet’	0.55 ± 0.05mn	23.14 ± 0.16mn	0.25 ± 0.00n	20.40 ± 4.44ef	0.06 ± 0.02j	0.10 ± 0.07n	44.50kl
‘Chelyabinka’	6.17 ± 0.27d	57.87 ± 1.34b	8.97 ± 0.08a	13.48 ± 0.28hij	1.60 ± 0.11cd	1.01 ± 0.06e	89.10cd
‘Chernichka’	4.84 ± 0.07f	39.28 ± 0.71h	4.13 ± 0.11jk	26.30 ± 0.16cd	2.62 ± 0.40b	1.43 ± 0.07d	78.60e
‘Dlinnoplodnaya’	9.95 ± 0.26c	49.66 ± 1.73d	7.30 ± 0.07c	25.62 ± 1.21cd	2.45 ± 0.43b	2.03 ± 0.39b	97.01b
‘Fialka’	0.86 ± 0.02m	58.47 ± 1.17b	8.23 ± 0.32b	9.73 ± 1.28klm	1.17 ± 0.33defg	0.59 ± 0.00ghi	79.05e
‘Goluboe Vereteno’	4.34 ± 0.20g	43.55 ± 2.26f	5.03 ± 0.15gh	10.68 ± 2.10ijkl	1.10 ± 0.01efgh	0.52 ± 0.02hij	65.22gh
‘Jolanta’	4.69 ± 0.16f	22.76 ± 0.14mn	3.22 ± 0.12l	11.25 ± 0.68ijkl	1.28 ± 0.10defg	0.76 ± 0.02fg	43.96kl
‘Kamchadalka’	5.45 ± 0.13e	60.37 ± 0.70a	5.97 ± 0.04e	17.31 ± 3.64fg	1.04 ± 0.47efgh	0.83 ± 0.16ef	90.96c
‘Karina’	4.24 ± 0.02g	25.09 ± 0.10l	3.44 ± 0.09l	12.55 ± 1.20ijk	1.46 ± 0.23cde	0.82 ± 0.07ef	47.60jk
‘Klon 38’	4.90 ± 0.11f	24.05 ± 0.43lm	3.45 ± 0.04l	9.71 ± 0.96klm	0.79 ± 0.09fgh	0.74 ± 0.03fg	43.64kl
‘Klon 40’	5.78 ± 0.12e	37.07 ± 0.46i	0.43 ± 0.01n	6.00 ± 0.54no	0.65 ± 0.07hi	0.12 ± 0.01mn	50.06j
‘Klon 44’	0.39 ± 0.04mn	17.24 ± 1.11o	2.30 ± 0.01m	6.00 ± 0.19no	0.78 ± 0.04fgh	0.39 ± 0.03ijkl	27.11n
‘Krupnoplodnaya’	2.60 ± 0.02j	41.56 ± 0.04g	0.31 ± 0.02n	14.08 ± 0.49ghi	0.05 ± 0.01j	0.08 ± 0.02n	58.68i
‘Kuvshinovidnaya’	3.65 ± 0.02h	31.35 ± 0.23k	0.16 ± 0.00n	4.92 ± 0.28no	1.02 ± 0.03efgh	0.20 ± 0.01lmn	41.30l
‘Leningradskii V.’	1.52 ± 0.12l	46.78 ± 0.24e	0.46 ± 0.02m	11.91 ± 0.70ijkl	0.25 ± 0.02ij	0.04 ± 0.00n	60.95hi
‘Morena‘	5.56 ± 0.20e	46.82 ± 1.69e	5.93 ± 0.55e	5.55 ± 0.20no	1.37 ± 0.03de	0.34 ± 0.01jkl	65.57g
‘Nympha’	4.22 ± 0.25g	47.70 ± 1.18e	4.65 ± 0.35hi	4.07 ± 0.09o	1.38 ± 0.03cde	0.22 ± 0.01klmn	62.24ghi
‘Roksana’	2.19 ± 0.12k	34.27 ± 0.65j	3.45 ± 0.32k	27.33 ± 0.33c	2.37 ± 0.51b	1.45 ± 0.13d	71.06f
‘Sineglazka’	5.72 ± 0.43e	51.64 ± 1.03cd	7.42 ± 0.12c	10.37 ± 0.68jklm	0.83 ± 0.03fgh	0.80 ± 0.07efg	76.78e
‘Tomichka’	10.48 ± 0.07b	52.77 ± 0.66c	8.41 ± 0.13b	37.39 ± 0.86a	3.21 ± 0.44a	3.23 ± 0.00a	115.50a
‘Vasilevskaya’	0.57 ± 0.01mn	51.54 ± 0.19cd	6.84 ± 0.23d	23.08 ± 1.11de	1.63 ± 0.10cd	1.66 ± 0.10c	85.32d
‘Viola’	12.57 ± 0.13a	25.63 ± 0.34l	2.40 ± 0.03l	16.21 ± 3.72gh	1.44 ± 0.05cde	0.72 ± 0.17fgh	58.99i
‘Volshebnica’	4.79 ± 0.26f	51.31 ± 0.08cd	5.72 ± 0.05e	14.27 ± 0.35ghi	0.99 ± 0.15efgh	0.95 ± 0.01ef	78.03e
‘Vostorg’	4.35 ± 0.08	35.31 ± 0.02g		8.40 ± 0.77lmn			48.07jk
‘Wojtek’	3.18 ± 0.03i	21.75 ± 0.03n	2.40 ± 0.04m	7.08 ± 0.45mno	0.79 ± 0.05fgh	0.44 ± 0.02ijk	35.63m

3-CQA, neochlorogenic acid; 5-CQA, chlorogenic acid; CQA, caffeoylquinic acid; di CQA 1, dicaffeoylquinic acid isomer 1; di CQA 2, dicaffeoylquinic acid isomer 2; di CQA 3, dicaffeoylquinic acid isomer 3; Total PA, total amount of quantified phenolic acids; ^1^ Values are expressed as the mean (n = 3) ± standard deviation. Mean values with different letters (a, b, c, etc.) within the same column are statistically different (*p* < 0.05). ^2^ ‘Bakcharskaya Y.’, ‘Bakcharskaya Yubileynaya’; ‘Leningradskii V.’, ‘Leningradskii Velikan’.

**Table 7:** Antioxidant activity (μmol TE/g fw) of different blue honeysuckle cultivars and genotypes.

Cultivar/Genotype	DPPH	FRAP
‘Amphora’	16.57 ± 1.89f ^1^	44.57 ± 1.30ef
‘Amur’	26.66 ± 1.77b	55.69 ± 1.47b
‘Atut’	8.80 ± 0.17n	22.86 ± 1.15s
‘Bakcharskaya Y.’ ^2^	13.53 ± 0.18i	33.51 ± 1.14lm
‘Berry Smart Blue’	11.70 ± 0.14klm	31.61 ± 0.92n
‘Blue Velvet’	18.50 ± 1.61e	28.23 ± 0.51op
‘Chelyabinka’	14.95 ± 0.44g	41.03 ± 1.86i
‘Chernichka’	11.43 ± 0.29lm	28.74 ± 1.64op
‘Dlinnoplodnaya’	15.80 ± 0.18fg	43.10 ± 1.37fg
‘Fialka’	16.40 ± 0.34f	42.72 ± 1.40gh
‘Goluboe Vereteno’	12.92 ± 0.49ij	35.07 ± 1.25kl
‘Jolanta’	27.30 ± 0.94b	52.77 ± 1.72c
‘Kamchadalka’	11.83 ± 0.84jklm	31.10 ± 0.90n
‘Karina’	8.94 ± 0.27n	22.41 ± 0.67s
‘Klon 38’	11,06 ± 0.32m	29,07 ± 1.24o
‘Klon 40’	28.77 ± 1.43a	57.52 ± 0.78a
‘Klon 44’	9.80 ± 0.67n	25.04 ± 1.10r
‘Krupnoplodnaya’	18.89 ± 0.82e	27.28 ± 1.33p
‘Kuvshinovidnaya’	26.48 ± 0.89b	50.41 ± 2.34d
‘Leningradskii V.’	18.90 ± 0.72e	31.52 ± 1.57n
‘Morena‘	15.59 ± 0.96fg	45.80 ± 0.92e
‘Nympha’	23.89 ± 0.31c	50.24 ± 1.54d
‘Roksana’	11.63b ± 0.19klm	32.06 ± 1.09mn
‘Sineglazka’	14.77 ± 0.33gh	41.32 ± 0.77hi
‘Tomichka’	13.74 ± 0.75hi	37.94 ± 1.32j
‘Vasilevskaya’	12.78 ± 0.86ijk	32.58 ± 1.47mn
‘Viola’	11.46 ± 0.84lm	28.14 ± 1.08op
‘Volshebnica’	12.29 ± 0.29jkl	31.35 ± 1.07n
‘Vostorg’	22.69 ± 1.62d	42.74 ± 1.78ghi
‘Wojtek’	19.54 ± 1.95e	35.52 ± 2.79k

^1^ Values are expressed as the mean (n = 3) ± standard deviation. Mean values with different letters (a, b, c, etc.) within the same column are statistically different (*p* < 0.05). ^2^ ‘Bakcharskaya Y.’, ‘Bakcharskaya Yubileynaya’; ‘Leningradskii V.’, ‘Leningradskii Velikan’.

## Supplementary Materials

Supplementary Materials are available online.
