# Supplementary material for: Iridoids, Phenolic Compounds and Antioxidant Activity of Edible Honeysuckle Berries (Lonicera caerulea var. kamtschatica Sevast.)
Source: Molecules. 2017 Mar 5;22(3):405. doi: 10.3390/molecules22030405 (PMC6155291; doi:10.3390/molecules22030405)
Supplement: Supplementary file 1 [file molecules-22-00405-s001.pdf]

molecules

Supplementary Materials: Iridoids, phenolic compounds and antioxidant activity of edible honeysuckle berries (*Lonicera caerulea* var. *kamtschatica* Sevast.)

Alicja Z. Kucharska, Anna Sokół-Łętowska, Jan Oszmiański, Narcyz Piórecki and Izabela Fecka

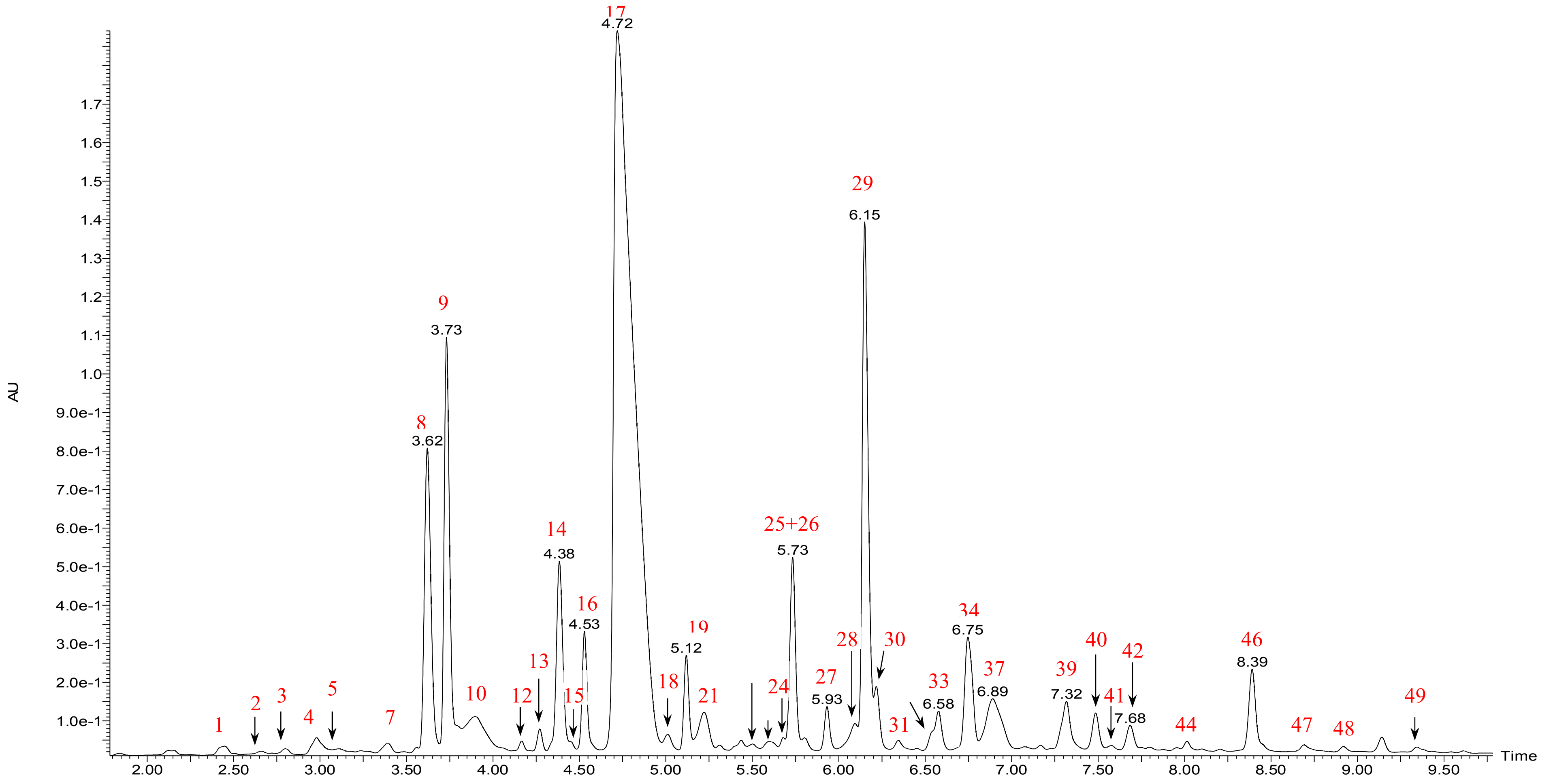

Fig. S1. UPLC-DAD chromatogram (254 nm) of compounds of methanolic extract from honeysuckle berries. The peak number corresponds to the number in Tables 1.

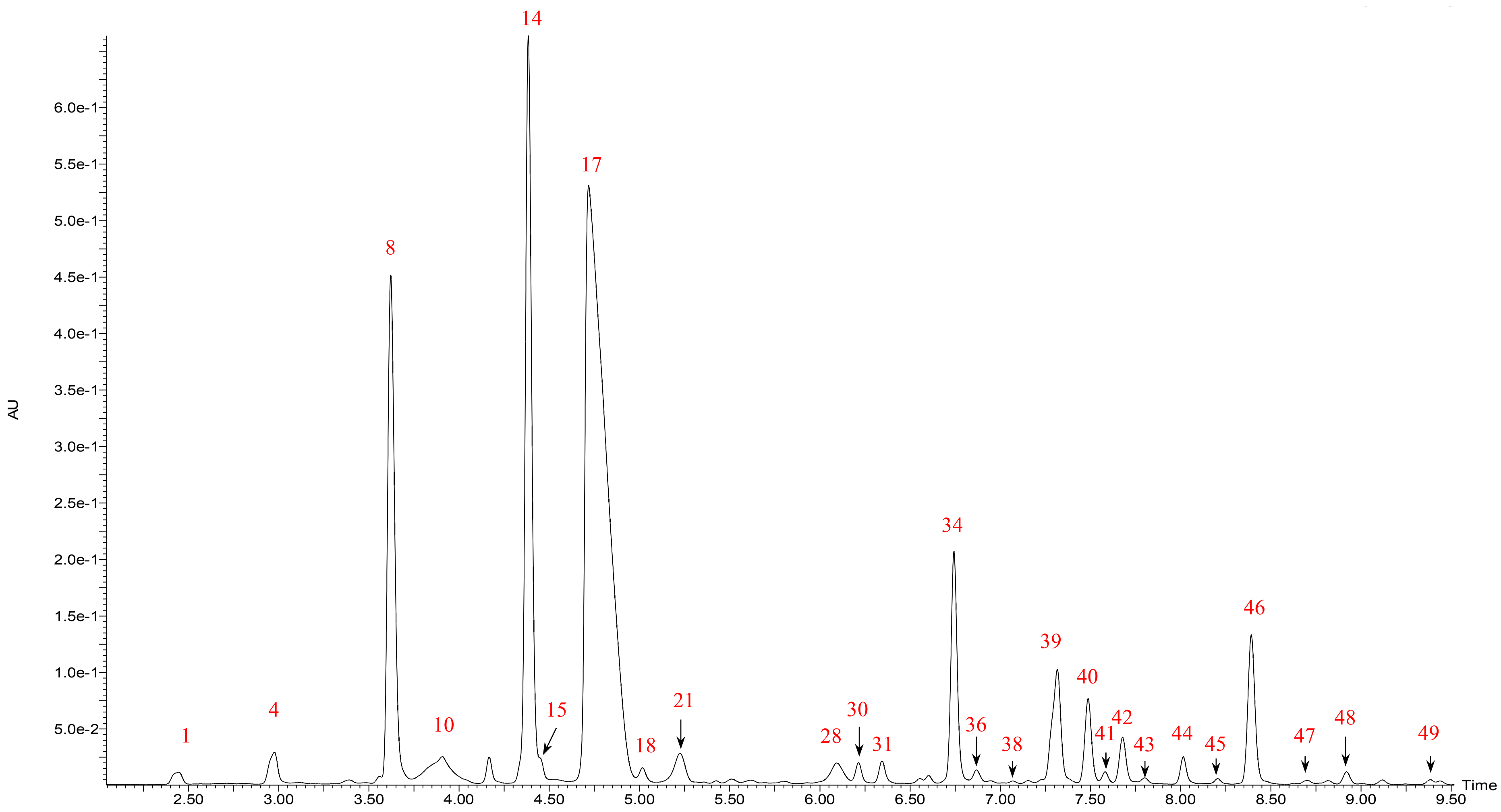

Fig. S2. UPLC-DAD chromatogram (360 nm) of compounds of methanolic extract from honeysuckle berries. The peak number corresponds to the number in Tables 1.

molecules
MDPI

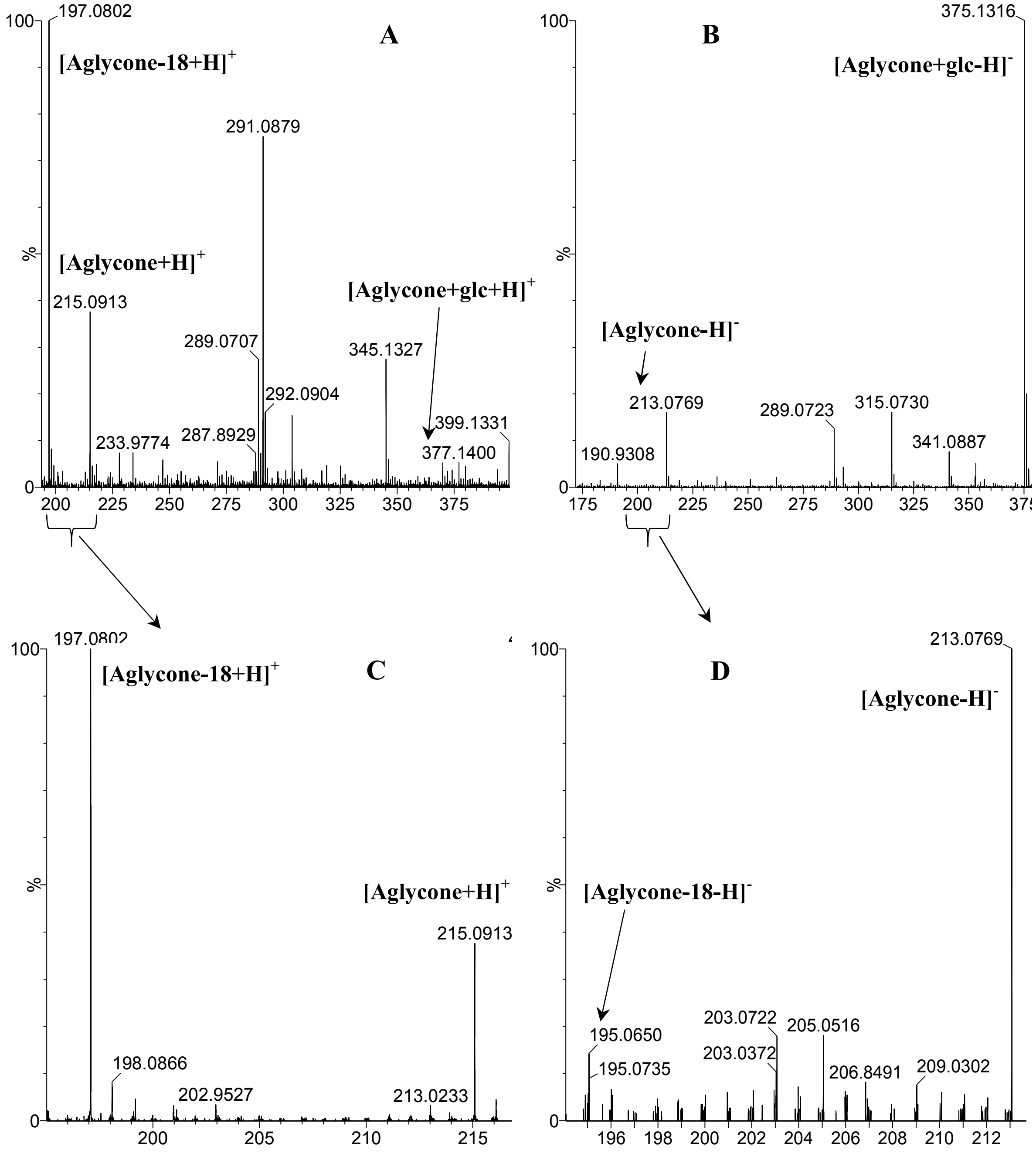

Figure S3. Mass spectra of the pseudomolecular and fragment ions of 8-*epi*-loganic acid (**3**) in positive (A, C) and negative (B, D) mode.

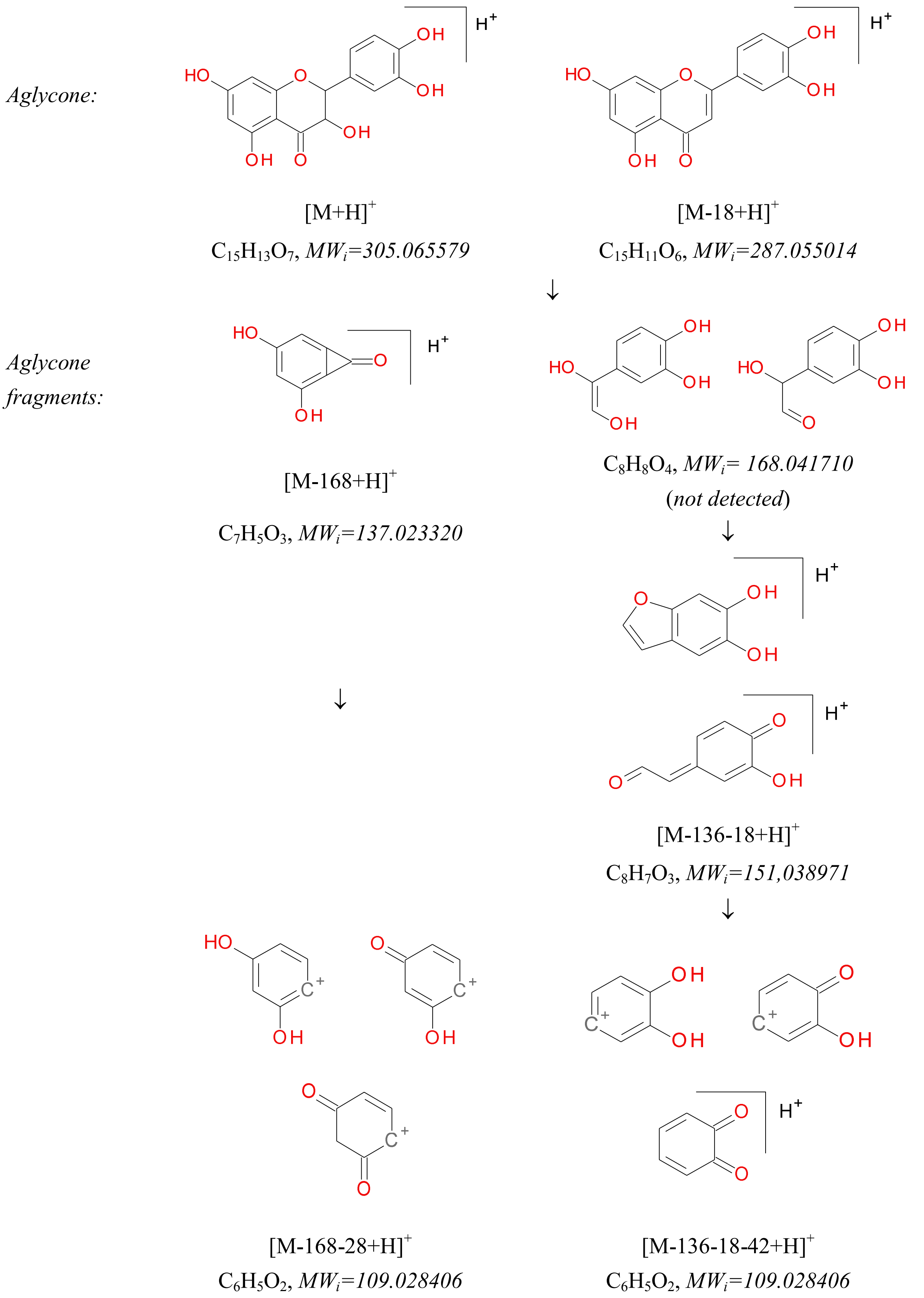

Figure S4. Structures of taxifolin glycosides fragment ions identified in positive mode (Assignation: *MWi*, monoisotopic molecular weight; -18, water; -28, C=O; -42, $H_2C=C=O$/ethenone).

Table S1. Mass errors of taxyfolin (aglycone) pseudomolecular ion and its fragment ions according to Figure S4

Formula [M+H]+	*MWi* detected [Da]	*MWi* calculated [Da]	Mass error [ppm]
$C_{15}H_{13}O_7$	305.0649	305.065579	2.2
$C_{15}H_{11}O_6$	287.0536	287.055014	4.9
$C_7H_5O_3$	137.0236	137.023320	2.0
$C_8H_7O_3$	151.0394	151.038971	2.8
$C_6H_5O_2$	109.0287	109.028406	2.7

Table S2. Correlation between main active compounds and antioxidant activity of blue honeysuckle berries.

	DPPH	FRAP
Total of iridoids	0.23	0.22
Total of anthocyanins	0.78	0.94

Table S3. Linear range, calibration curve, correlation coefficient R, LOD, and LOQ data for six used standards.

Standard	Linear range (µg/mL)	λ_{det}[1] (nm)	Calibration curve	Correlation coefficient *R*	LOD (µg/mL)	LOQ (µg/mL)
Cyanidin 3-*O*-glucoside	10 – 75	520	y=1.204x-0.931[2]	0.9995	0.086	0.283
Quercetin 3-*O*-glucoside	20 – 150	360	y=0.606x-1.687	0.9995	0.060	0.185
Luteolin 7-*O*-glucoside	20 – 150	360	y=0.928x+1.468	0.9996	0.041	0.135
5-*O*-Caffeoylquinic	20 – 300	320	y=1.083x+0.819	0.9999	0.060	0.199
Loganic acid	20 – 300	245	y=0.361x+0.341	0.9998	1.748	5.769
Loganin	20 – 200	245	y=0.438x+0.743	0.9998	1.168	3.853

[1] λ_{det} detection wavelength in quantification process.

[2] y - peak area, x - concentration.
